# Predicting merchant future performance using privacy-safe network-based features

**DOI:** 10.1038/s41598-023-36624-0

**Published:** 2023-06-21

**Authors:** Mohsen Bahrami, Hasan Alp Boz, Yoshihiko Suhara, Selim Balcisoy, Burcin Bozkaya, Alex Pentland

**Affiliations:** 1 MIT Connection Science, Institute for Data, Systems, and Society, Massachusetts Institute of Technology, Cambridge, MA 02139 USA; 2 Faculty of Engineering and Natural Sciences, Sabanci University, Istanbul, Turkey; 3 MIT Media Laboratory, Massachusetts Institute of Technology, Cambridge, MA 02139 USA; 4 Sabanci Business School, Sabanci University, Istanbul, Turkey

**Keywords:** Socioeconomic scenarios, Computational science

## Abstract

Small and Medium-sized Enterprises play a significant role in most economies by contributing to job creation and economic growth. A majority of such merchants rely on business financing, and thus, financial institutions and investors need to assess their performance before making decisions on business loans. However, current methods of predicting merchants’ future performance involve their private internal information, such as revenue and customer base, which cannot be shared without potentially exposing critical information. To address this problem, we first propose a novel approach to predicting merchants’ future performance using credit card transaction data. Specifically, we construct a merchant network, regarding customers as bridges between merchants, and extract features from the constructed network structure for prediction purposes. Our study results demonstrate that the performance of machine learning models with features extracted from our proposed network is comparable to those with conventional revenue- and customer-based features, while maintaining higher privacy levels when shared with third-party organizations. Our approach offers a practical solution to privacy concerns over data and information required for merchants’ performance prediction, enabling safe data-sharing among financial institutions and investors, helping them make more informed decisions on allocating their financial resources while ensuring that merchants’ sensitive information is kept confidential.

## Introduction

Small and Medium-sized Enterprises (SMEs) constitute a significantly large portion of economies around the world and thus play a vital role in economic stability and growth. According to the Annual Report on European SMEs 2021/2022^1^, 99.8% of European enterprises consist of SMEs, which account for 65% of total employment in the business economy. The U.S. Small Business Administration (SBA) reported that SMEs were responsible for employing 61.7 million people, which is equal to 46.4% of the total employed workforce in the private sector in 2022^2^. These statistics indicate that the success and failure of SMEs can directly affect the levels of (un)employment in countries.

On the other hand, according to the U.S. Bureau of Labor Statistics, between 1994 and 2019, about 33% of the newly opened SMEs failed within their first two years, and only roughly 50% of them survived after their first five years^3^. Their historical records indicate that the SME failure and default rates can drastically worsen during economic downturns and financial crises. Considering the importance of SMEs for economic stability, it is of crucial importance for governments, responsible organizations, and authorities to support and oversee the temporal performance and well-being of SMEs.

Moreover, most SMEs heavily rely on business financing from banks and/or other financial institutions and entities to run their businesses^4^. However, given the noticeable failure rates of SMEs, financial institutions and investors have to carefully assess SMEs to make decisions on business loans. Such institutions and entities seek methodologies that provide insights into the determinants of success, creditworthiness, and distress level of an SME. Therefore, an accurate well-being assessment and performance prediction is of critical importance for financial institutions as they determine the overall risk level and leverage position for these institutions, and potentially the entire financial ecosystem.

However, current methods of predicting future performance of SMEs, involve their private internal information, such as revenue and customer base, which cannot be shared without potentially exposing critical information. On the other hand, SMEs are typically involved in resource-constrained operations that suffer from the scarcity of certified financial statements and publicly available information on debt, equity, or liquidity^5–7^. Additionally, unlike public companies that publish financial reports periodically, SMEs are not obligated to publish their financial activity reports, and thus, it is not feasible for financial institutions to monitor and track the financial activities and positions of every single SME.

Given the highly competitive environments that SMEs operate in, they always have privacy concerns about sharing their internal information with other firms, which could harm their position in case of leakage to their competitors. Therefore, the scarcity or unavailability of SMEs’ internal information and financial records is a big hurdle in replicating the models built on such data. On the other hand, sharing data with third-party organizations has always been a delicate matter for financial institutions. In such cases, legally binding but also time demanding non-disclosure agreements (NDA) between entities help data to be secured to a certain extent.

This study endeavors to examine the SME performance evaluation and prediction (also referred to as “merchants” hereafter) while addressing concerns regarding data privacy and data sharing safety. We aim to achieve these objectives without directly accessing the internal financial metrics of the merchants, instead, by leveraging a comprehensive dataset of credit card transactions on a large scale.

First, in order to evaluate a merchant’s performance level, we present a new assessment measure as a function of the relative changes in future transactions, revenue, and customers of a merchant. Subsequently, we propose to derive a feature set from credit card transaction data instead of the internal financial metrics of merchants. Thus, banks can easily incorporate the approach presented here into their decision support models. We refer to these features as *revenue-based features*. Then we extract customer information to answer the question of ‘who visits the shop?’. The features extracted from customer information are referred to as *customer-based features*. The revenue-based and customer-based feature sets will serve as the baselines for our analyses.

Next, we propose a novel network model of merchants based on customer credit card transaction patterns and investigate merchants’ positions in the network using network centrality metrics namely: node degree, betweenness, closeness, and eigenvector centrality. In addition to these centrality metrics, we obtain diversity variables using the ego-network of each merchant to capture its surroundings. These features are referred to as *network-based features*. We further use the proposed network structure for learning continuous numeric feature representations for nodes (i.e., merchants) in the network (node2vec)^8^. We refer to these features as *node2vec features*.

Finally, we extract a set of features based on merchants’ available information for banks and financial institutions. These features include information about merchants’ business categories, socio-demographic information about their neighborhood residents, and physical factors including the number and diversity of other businesses and amenities in their vicinity. These *merchant-based features* are utilized in all of our analyses in addition to the other four extracted feature sets (i.e., revenue-based, customer-based, network-based, and node2vec feature sets). The resulting feature sets are fed into different types of machine learning models to predict merchants’ future performance levels.

The results of these analyses show that the proposed network-based features’ performance is comparable to those of conventional revenue-based and customer-based features, indicating that the proposed merchant network is effective in capturing the performance level of merchants and offering promising results for future studies. The proposed network-based and node2vec features possess a crucial attribute: not only do they offer valuable insights into the present and future performance of merchants, but they also ensure a significantly enhanced level of privacy protection compared to raw financial records. Therefore, these features can be safely shared with third-party organizations in a structured tabular format. This emphasis highlights the importance of these features in striking a balance between providing valuable insights and preserving merchants’ privacy while facilitating secure data sharing with external entities.

To summarize, the contributions of this paper are as follows:

- We introduce a new approach to defining the performance level of merchants and verify that the labels do not exhibit any particular biases concerning the geographical locations, income levels of the region’s residents, or the socio-economic status of the merchants’ customers.
- We propose constructing a merchant network based on customer purchase transaction records. We then leverage the four commonly used network centrality scores (i.e., node degree, betweenness, closeness, and eigenvector centrality.) and diversity variables obtained from their ego networks as input features to predict their future performance.
- We conduct our analysis on a large-scale credit card transaction dataset and show that the predictive models with our proposed network-based and node2vec features yield comparable performance to those using conventional features sets (i.e., revenue- and customer-based) while ensuring privacy and safeguarding any sensitive information regarding merchants, and thus, enabling secure data sharing with third-party organizations.

The methods and approaches outlined in this research are poised to pave the way for the advancement of methodologies enabling businesses and financial entities to exchange the valuable insights within their data, while preserving the confidentiality of the raw data.

The remainder of this paper is outlined as follows. We commence by examining the current state-of-the-art literature, which serves as a foundation for the methodologies that have influenced our study. Next, we introduce our proposed methodology, describe the datasets employed in this research, and delineate the analytical setting. Subsequently, we present a comprehensive analysis of our results. Finally, we engage in a discussion regarding the implications derived from our findings and offer concluding remarks to wrap up the paper.

## Background

Machine learning constitutes the backbone of the majority of merchant well-being and performance prediction studies^9–12^. Dominant merchant performance assessment methodologies involve quantitative risk models that combine various financial metrics such as earnings per asset, equity per asset, and debt ratio^13–15^. Gallucci et al.^16^ incorporate financial metrics, bank-firm interaction information, and corporate governance variables in SME loan default prediction using a Bayesian model. Features extracted from financial statements of corporations, such as debt ratio, total capital turnover and quick ratio, have been used by Son et al.^17^ on a Gradient Boosting framework. To predict business failure, Kim et al.^12^ make use of the tree-based majority voting ensemble method which includes, in addition to the financial features, the recession indicator computed by the National Bureau of Economic Research (NBER) as a macro-economic feature to account for the overall economic status.

Other research incorporates an SME’s principal owner’s credit information in the quantitative risk model in order to project the risk for the SME^18^. This approach seeks to tackle the fundamental lack-of-data issue underlying SME risk scoring; however, such methods still involve exclusively collected data. This data deficiency has led the researchers to explore potential substitute data and proxies. Another recent study^19^ relies on web mining to extract proxy features from online resources, such as TripAdvisor and OpenStreetMap for predicting SME growth for restaurants in Switzerland where revenue information is known a priori.

To account for local economies’ effect on SMEs, Fernandes and Artes^20^ propose a new variable based on ordinary kriging to help assess the risk of credit default among SMEs. The proposed variable was able to enhance the predictive power of the logistic model on credit scoring. Yoon and Kwon^6^ have shown that credit card data can be highly informative about the financial position of SMEs. They built a support vector machines (SVM) model to predict bankruptcies, using the variables such as sales fluctuation and sales patterns extracted from credit card transactions.

Inspired by social physics^21,22^ and computational social science ^23^, which take data-driven approaches toward understanding human behavior, we take a similar approach to understand merchant performance. We go one step beyond the current stage of bankruptcy prediction by using a large-scale credit card transaction dataset and leveraging machine learning models to predict merchants’ future performance without directly accessing their internal financial metrics.

Building upon the findings of a previous study^24^, which highlighted the significance of social bridges in comprehending the purchasing behaviors across diverse communities, we leverage customers as bridges between merchants in order to gain deeper insights into the future performance of the merchants. This approach recognizes the influential role of social connections in shaping economic dynamics and allows us to explore the potential of network science in understanding social phenomena.

Furthermore, a substantial body of literature in social physics has employed network science to enhance our understanding of various social phenomena. Studies in this domain have consistently demonstrated that interactions and information exchange through networks can promote productivity^25–30^. It is widely recognized that networks serve as the fundamental framework for social and economic activities, underpinning the fabric of society^31^. By integrating these insights from network science into our study, we can shed light on the intricate dynamics of social and economic interactions, providing a comprehensive understanding of merchant performance.

As we live in a social network, merchants run their businesses in a merchant network as well; therefore, we contend that the structural properties of merchants in a network are essential to their performance prediction. To this end, we propose constructing a *merchant network*, using credit card transaction data, with the premise that customers can act as bridges between merchants. We use network centrality metrics (i.e., degree and strength, betweenness, closeness, and eigenvector centrality) as indicators of merchants’ performance in the network. The merchant network should enable us to capture the positioning of merchants by incorporating their surrounding information. We hypothesize that the network centrality metrics in the merchant network can potentially provide holistic signals for better understanding and predicting merchants’ future performance .

## Methods

In this section, we introduce the utilized credit card dataset and its basic statistics. We then describe our methodology and the analytical setting for evaluating the performance of our proposed methods.

### Data and pre-processing

In this study, we use a credit card transaction dataset from a major bank in an OECD country^32^. The sampled dataset consists of three tables, namely: customer, merchant, and transaction tables.

The customer table contains an anonymous customer ID, and customer demographic information including customer age, gender, marital status, education level, estimated income, and home and work district IDs. Districts are administrative areas within the metropolitan area of study, and each district is governed by a local municipality. Districts have an average size of 150 square kilometers and an average population of 380,000 residents.

The merchant table contains anonymous merchant ID, merchant ISO 18245 category code (MCC)^33^, and merchant district ID. Merchants may belong to various categories such as grocery stores and supermarkets, restaurants, gas stations, clothing stores, and bookstores, which are described by their MCCs.

The transaction table contains transaction records of customers, including merchant ID, customer ID, the amount of the transaction, and the timestamp. The original dataset consists of 4,507 merchants, 62,194 customers, and 2,511,527 transactions over the span of one year from July 2014 to July 2015. More information about the tables described above is provided in “Supplementary Information”.

Since our aim is to predict future performance level for private sector merchants, we remove non-discretionary MCCs such as government-owned places, parking lots, lodging, and other similar categories. Then, to ensure the robustness of the analyses, we filter out merchants that recorded less than 10 transactions per month in each period. A period can be equal to one or more months based on the amount of available data. More detailed information is provided in the section Analytical setting.

Finally, we only keep the MCCs that contain more than 10% of remaining merchants in the dataset, and remove the districts with less than 10 merchants remaining. As a result, the pre-processed data contains 1,977 merchants in 3 MCCs. Table 1 shows the number of remaining merchants in each MCC after data pre-processing.

**Table 1:** Business categories ordered by their merchant counts after data pre-processing.

Description	MCC	Merchant count
Grocery stores, supermarkets	5411	1118
Men’s and women’s clothing stores	5691	464
Service stations (with or without ancillary services)	5541	395

The pre-processed dataset contains no information that could potentially be used to identify individual customers. A complete list of business categories and the related numbers of transactions and merchants are provided in “Supplementary Information”.

### Methodology

In this section, we initially establish the merchant network concept and provide a detailed explanation on its construction using customer credit card transactions. Then, we describe merchant network features that capture signals of merchants’ performance in a holistic manner. Finally, we delineate the analytical setting for evaluating the method’s performance in comparison to baseline methods.

#### Merchant network

In recent years, there has been a growing recognition of the ability of networks and their dynamics to capture distinctive characteristics of various phenomena. Generally, networks characterizing a certain phenomenon exhibit inherent suitability for making inferences about the future state of entities (nodes) in those networks. As previous studies have shown^34,35^, structural properties of nodes in a network are capable of encoding the capacity for improvement for the nodes; hence, networks are particularly well-suited for applications that involve investigating and making inferences about the future of specific entities^36^. Inspired by those findings, using the credit card transaction dataset we define and form a merchant network as follows.

**Figure 1:**
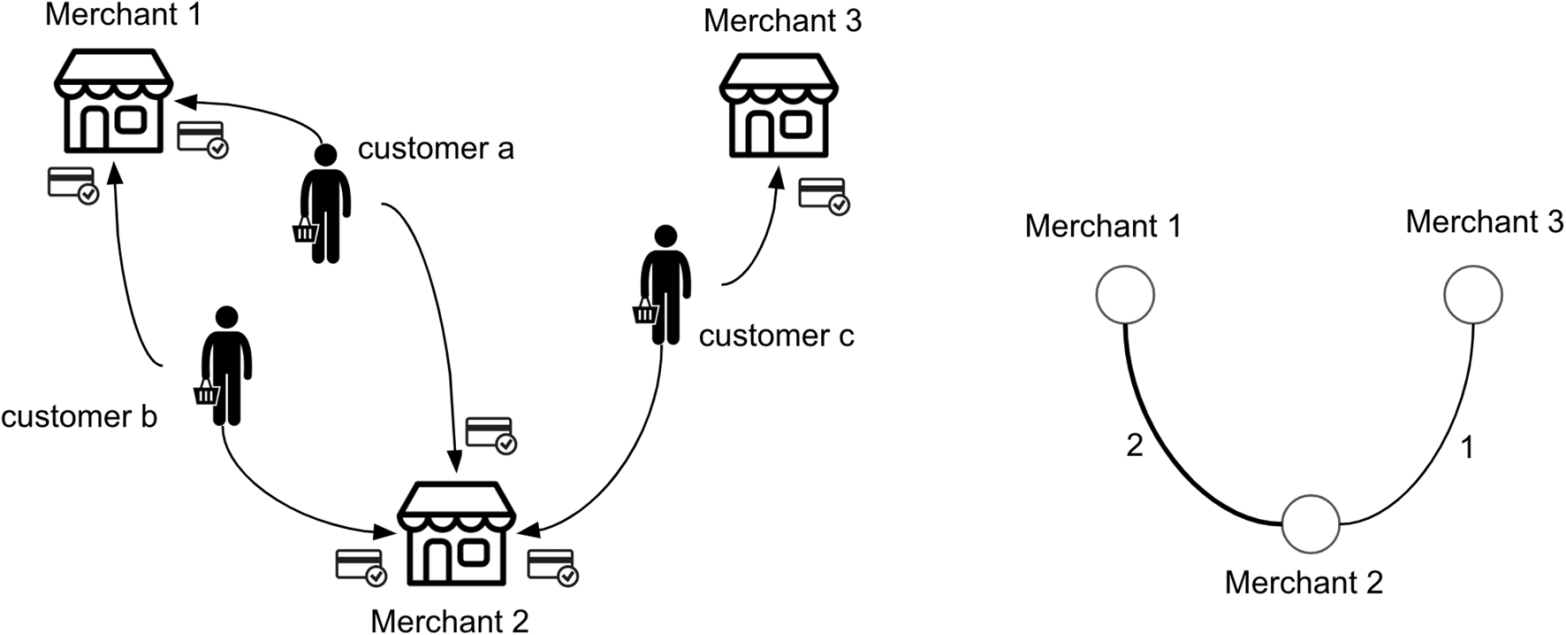
(Left) Example of customer transactions at merchants. (Right) Merchant network extracted from the transactions.

Let $v_i \in V$ denote a vertex for merchant *i* (shown by $m_i$) and $e_{ij}\in E$ is an edge between vertices $v_i$ and $v_j$. A merchant network is an undirected graph $G = (V, E, W)$, where the weight $w_{ij} \in W$ is defined as:

1 $$\begin{aligned} w_{ij} = \left| \left\{ c_k | \exists (c_k, m_i) \in {\mathscr {D}} \wedge \exists (c_k, m_j) \in {\mathscr {D}}, c_k \in {\mathscr {C}}\right\} \right| \end{aligned}$$

where $\mathscr {D}$ is credit card transaction data and $\mathscr {C}$ is the set of all customers. The edge weight $w_{ij}$ is defined by the number of distinct customers who made purchases at both merchants *i* and *j*, and we define neighbors (also referred to as alters) of merchant *i* as the set of merchants that are directly connected to merchant *i* in graph *G* and denoted by $A_i$. Figure 1 depicts an example of how a merchant network is created from credit card transactions. Intuitively, the weight of an edge between two merchants increases as more customers purchase at both merchants.

#### Merchant network-based features

In order to extract signals specific to each merchant from a constructed merchant network, it is necessary to aggregate information within each node of the network. In this paper, we consider using the four most commonly employed centrality metrics (i.e., degree and strength, betweenness, closeness, and eigenvector centrality) and two diversity metrics as the signals of merchant current and future performance level. Each merchant’s importance is measured by four centrality metrics based on its position in the network. Those metrics are known to capture different perspectives on the importance of a node^37^. In what follows, we introduce those centrality measures and interpret what each of those measures may mean in our proposed merchant network. We also derive two diversity measures from the proposed network structure to understand a merchant’s capability in attracting customers from different areas and with different preferences and needs.

##### Degree and strength^38^

are computed based on a node’s direct ties with other nodes in a network. Degree of a node is equal to the number of its direct ties with other nodes, which is also equal to the number of alters of merchant *i* as defined to be members of the set $A_i$. Node strength is equal to the sum of the weights of edges that the node shares with others. Here, the strength of a node (i.e., merchant) is identical to the number of distinct customers in common with the other merchants in the network. Merchants’ degrees and strengths are well correlated with their revenues during each corresponding period of time.

##### Betweenness centrality^38^

is a measure of how important a node is based on the shortest paths through the network. It can also be seen as the extent to which a node lies on the shortest path between other nodes in the network. This measure takes into account the connectivity of the node’s neighbors, giving a higher value for nodes which bridge clusters of nodes. In our case, it can capture the number of merchants to which a shop is connecting indirectly through their direct links^39^.

##### Eigenvector centrality^40^

is an extension of a node’s degree centrality. This centrality metric measures a node’s importance while giving consideration to the importance of its neighbors^41^. In our network, it roughly encodes the probability that a random customer will visit a particular merchant.

In addition to the centrality measures, we also consider two ego network diversity metrics using the constructed merchant network to capture the dynamics of a node’s interactions with different geographies and business categories.

##### Closeness centrality^38^

is the extent that a node is close to other nodes in a network. It is a proxy of measuring the ability of a merchant to access information of other merchants in its network through its customers.

##### **Geographical diversity**

measures the geo-spatial diversity of neighbors of the target merchant *i*. We denote this variable with $D_{g}^{i}$ and calculate the geographical diversity based on the geographical distributions of the neighbor merchants of a merchant. Specifically, we use the district information to compute the probabilistic distribution of the districts. To quantify the diversity, we utilize Shannon entropy ^42^, a widely adopted metric in this context ^43^. The formalization of Shannon entropy for computing the geographical diversity is depicted by Eq. (2).

2 $$\begin{aligned} D_{g}^{i} = \sum _{h \in I_{H}^{i}} - p_{h}^{i} \log {p_{h}^{i}} \end{aligned}$$

where $p_{h}^{i}$ is the fraction of the edge weights of merchant *i* connected to other merchants from district *h* over all districts having edges with merchant *i* denoted by $I_{H}^{i}$.

##### Business-category diversity

measures the diversity of the business categories of neighbors of merchant *i*. Similar to the geographical diversity, we consider the business-category diversity of a merchant’s ego network with respect to the business category of its neighbors. We formalize this metric with $D_{c}^{i}$ and use the following equation to calculate it.

3 $$\begin{aligned} D_{c}^{i} = \sum _{b \in I_{B}^{i}} - p_{b}^{i} \log {p_{b}^{i}} \end{aligned}$$

where $p_{b}^{i}$ shows the fraction of the edge weights of merchant *i* connected to other merchants from business type *b* over all business types having edges with merchant *i* denoted by $I_{B}^{i}$.

#### Label definition

The main objective of this section is to establish a performance evaluation metric that can effectively gauge a merchant’s performance in a competitive environment, and at the same time can be derived from its transactional data. Prior research has employed a range of objective indicators (e.g., financial metrics) and subjective indicators (e.g., managers’ perceptions) to propose various performance measurements for SME merchants^44^. However, accessing a firm’s internal goals and its management’s perception of performance may not always be feasible, and even if attainable, there is no direct method for translating such information into a metric that can accurately indicate the firm’s market position and relative standing among competitors. Therefore, in this study, we leverage objective indicators, encompassing merchants’ sales, attractiveness to passers-by, and customer relationship information, to define a new performance metric.

Considering the magnitude and temporal scope of our dataset, along with the outcomes of preliminary analyses, we establish a period duration of 6 months, dividing the data records into two equal-length periods. Subsequently, we juxtapose a merchant’s revenues, number of unique customers, and number of transactions during the initial 6-month observation period with corresponding measures in the subsequent 6 months. We then calculate the rate of change for each metric using Eqs. (4–6).

4 $$\begin{aligned} \Delta {R}_{t+1,t}^{i}= & {} (R_{t+1}^{i} - R_{t}^{i}) / R_{t}^{i} \end{aligned}$$

5 $$\begin{aligned} \Delta {C}_{t+1,t}^{i}= & {} (C_{t+1}^{i} - C_{t}^{i}) / C_{t}^{i} \end{aligned}$$

6 $$\begin{aligned} \Delta {N}_{t+1,t}^{i}= & {} (N_{t+1}^{i} - N_{t}^{i}) / N_{t}^{i} \end{aligned}$$

where $R_{t}^{i}$, $C_{t}^{i}$, and $N_{t}^{i}$ denote the revenue, number of unique customers, and number of transactions of merchant *i* in period *t* (here we use the first 6 months) and $R_{t+1}^{i}$, $C_{t+1}^{i}$, and $N_{t+1}^{i}$ denote the revenue, number of unique customers, and number of transactions of merchant *i* in period $t+1$ (the remaining 6 months). It is important to note that the revenue of a merchant is calculated by aggregating the spending amount of all transactions made at the merchant in the corresponding period. Then we compare the rates resulting from Eqs. (4–6) for merchant *i* with the median value of the same indicators’ rates over all merchants in the same business category (MCC) as merchant *i*’s. By contrasting the rate of change in these metrics among merchants within the same MCC, one can significantly mitigate the impact of seasonality. This becomes particularly crucial when the available historical data is insufficient for capturing the full extent of seasonality effects.

Subsequently, for each rate of change, we label the merchants with rates above the median with 1 and those with rates below the median with 0 as binary-class labels using Eqs. (7–9). This kind of binary labeling is a common practice in the literature^45^.

7 $$\begin{aligned}{} & {} {I_{R}^{i}(t+1)} \rightarrow {\left\{ \begin{array}{ll} 1\quad if\quad \Delta {R}_{t+1,t}^{i}\; \ge \; median(\Delta {R}_{t+1,t}^{b_i})\\ 0\quad otherwise \end{array}\right. } \end{aligned}$$

8 $$\begin{aligned}{} & {} {I_{C}^{i}(t+1)} \rightarrow {\left\{ \begin{array}{ll} 1\quad if\quad \Delta {C}_{t+1,t}^{i}\; \ge \; median(\Delta {C}_{t+1,t}^{b_i})\\ 0\quad otherwise \end{array}\right. } \end{aligned}$$

9 $$\begin{aligned}{} & {} {I_{N}^{i}(t+1)} \rightarrow {\left\{ \begin{array}{ll} 1\quad if\quad \Delta {N}_{t+1,t}^{i}\; \ge \; median(\Delta {N}_{t+1,t}^{b_i})\\ 0\quad otherwise \end{array}\right. } \end{aligned}$$

Then for each merchant, we sum those three binary indicators (Eq. 10). If they sum up to 3–that is, the merchant’s performance is better than the median in all three indicators (i.e., change in revenue, unique customer count, and transaction count)–we label the merchant as a ‘well-performing’ merchant. These merchants can be considered as low-risk merchants since they have performed better than at least half of their counterparts. On the other hand, if all indicators for a merchant are equal to 0, it means that the merchant is under-performing in all indicators considering its competitors in the same MCC. Those merchants can be considered as high-risk merchants for investment and we label such merchants as ‘poorly-performing’ merchants. The remaining merchants are labeled as ‘medium-performing’ with medium risk level for financial institutions, and thus, in total, we have three performance classes. Equation (11) formalizes the last step in the labeling task.

10 $$\begin{aligned}{} & {} { I_{S}^{i}(t+1) = I_{R}^{i}(t+1) + I_{C}^{i}(t+1) + I_{N}^{i}(t+1)} \end{aligned}$$

11 $$\begin{aligned}{} & {} {L^{i}(t+1)} \rightarrow {\left\{ \begin{array}{ll} \text {`well-performing'}\qquad \qquad \quad if\quad I_{S}^{i}(t+1) = 3\\ \text {`medium-performing'}\qquad \quad \;\; if\quad I_{S}^{i}(t+1) = 2\;\; \text {or}\;\; I_{S}^{i}(t+1) = 1 \\ \text {`poorly-performing'}\qquad \qquad \; if\quad I_{S}^{i}(t+1) = 0 \end{array}\right. } \end{aligned}$$

#### Analytical setting

In this study, we use machine learning techniques to evaluate the effectiveness of the signals extracted from our proposed merchant network. In such evaluation frameworks that are based on supervised learning methods, one needs to derive input features and define the output labels. Our analytical setting is explained in what follows.

##### Feature extraction

In order to assess the effectiveness of the network-based feature set (Network), we conduct a comparative analysis with two baseline feature sets: revenue-based features (Revenue) and customer-based features (Customer). Previous research by Yoon and Kwon^6^ has demonstrated the viability of revenue-based features as a substitute for internal financial data in predicting merchant bankruptcy. Furthermore, studies such as those by Anderson et al.^46^ and Simester et al.^47^ have utilized customer-based features to forecast the success and failure of new products.

In addition to the features extracted from the credit card transaction data, we include the features that pertain to a merchant’s business category (i.e., MCC), its physical surrounding, and neighborhood attributes. Based on merchants’ locations, we extract the population and average household income of the districts they are located in. It is important to note that as shown by previous studies^29,48,49^, the number and diversity of points-of-interest (POIs) and amenities in a merchant’s proximity can increase its attractiveness for transient customers and improve its market potential. To this end, we use a dataset of POIs, provided by Here.com (a digital map production company), in which the POIs are grouped into twelve categories, namely: business centers, community service centers, financial institutes, educational institutes, entertainment places, shopping places, restaurants, hospitals, parks, travel destinations, auto services, and transportation hubs.

By leveraging the POI dataset and the geographic information of merchants, we examine the POIs situated within a 200-meter radius of each merchant. Subsequently, we calculate the quantity and category diversity of these nearby POIs. We refer to these features as merchant-based features (Merchant), as presented in Table 2. These features are incorporated as a fixed subset of input features into all models throughout the analyses.

The customer-based features are derived from the data related to individuals who have conducted transactions with the merchants included in our study. Some merchants target a specific demographic group of customers and some attract a wide range of demographic groups. By adding such features, we aim to take into account the attractiveness of each merchant with respect to the variety of customers. These features help us understand whether the merchants of interest are successful in attracting their target customers from different demographic groups and regions. The set of customer-based features is shown in Table 2. On the other hand, an existing study has used revenue-based features for bankruptcy prediction^6^. The proposed model uses historical records of a merchant’s sales, revenue, and customer profitability statistics. Here, we use the revenue-based features presented in Table 2.

The Network features are the main contribution of this study. We compute the network features based on a merchant network constructed for the first 6 months. As explained in the previous section, we extract the features shown in Table 2, including 4 centrality metrics and 2 diversity metrics based on a node’s position in the network structure. It is worth noting that although there are alternative centrality metrics available (e.g., PageRank), we chose not to incorporate them into the network-based feature set as they did not contribute to our analysis results.

Lastly, as a privacy-enhanced alternative to Network features, we employ features generated through representations of nodes utilizing the proposed network structure and topology. This is accomplished by using the node2vec algorithm^8^. We generate a feature vector of 128 dimensions for each merchant with return parameter *p* and in-out parameter *q* set to 1.3 and 1.2 respectively. Although it is possible to adjust all dimensions and hyperparameters of this technique, and several methods have been proposed to identify the optimal dimensions^50^, this study focuses on a different aspect. Instead of exploring and implementing optimization approaches, we adopt a commonly used practice of comparing the performance of various dimension choices (e.g., 50, 100, 128, 200, and 300) in the downstream task of predicting merchants’ future performance.

It is important to highlight that all feature sets are exclusively derived from credit card transactions within the initial 6 months (i.e., observation period). This strict selection ensures that no information from the prediction period is incorporated into the predictive models, thus maintaining their integrity.

**Table 2:** Feature sets employed as predictive variables and input for machine learning models in this study.

Feature sets			
Merchant	Customer	Revenue	Network
- Merchant MCC	- Number of unique customers	- Period total revenue	- Degree
- District population	- Age mean	- Number of transactions	- Closeness centrality
- District’s average per month household income	- Income mean and median	- Number of unique customers	- Eigenvector centrality
	- Education diversity		- Betweenness centrality
- Surrounding POI count	- Gender diversity		- Ego network geo diversity
- Category diversity of surrounding POIs	- Marital status diversity		- Ego network MCC diversity
	- Employment type diversity		
	- Home district diversity		
	- Work district diversity		
	- Distinct home district count		
	- Distinct work district count		

##### Machine learning algorithms

We use four machine learning models ^51^ built on four different types of algorithms in the analyses, namely: Multi-class Logistic Regression (LR), Support Vector Machines (SVM), Random Forest (RF), and Naive Bayes (NB). These algorithms are among the common choices for linear and non-linear models and major machine learning algorithms for a wide variety of domains. These methods are widely used in the bankruptcy prediction literature.

##### Evaluation metric

Following the standard machine learning evaluation framework , we conduct ten-fold cross-validation for classification and report area under the receiver operating characteristic curve (AU-ROC)^51^ as an evaluation metric for model performance. AU-ROC is a measurement of how well the classification models can distinguish between different classes. In our study case, since the labels are not severely imbalanced, AU-ROC is considered to be a suitable evaluation metric. In this setting, to account for multi-class labels, we use the One versus Rest (OVR) evaluation metric where AU-ROC is calculated for each class against the rest, and resulting scores are averaged. During cross-validation, we compute AU-ROC on test-fold and obtain mean and standard deviation across folds.

## Results

For the purpose of conducting our analyses, we construct a merchant network by utilizing customer co-purchase (edge) information extracted from the credit card transaction dataset. Subsequently, we compute the previously introduced centrality and diversity features from the derived network for each individual merchant (node). Next, we use our proposed performance evaluation metric for labeling the merchants according to their rank among their counterparts in the same MCC considering the rate of change in their revenue, number of transactions, and number of unique customers from one period to another. Finally, utilizing the extracted feature sets (e.g., network-based features), along with the performance labels, we leverage different machine learning methods to evaluate the performance of different features and feature sets in predicting the future performance of merchants.

### Label analysis

Among the studied 1,977 merchants, 590 (29.84%) are labeled as well-performing, 818 (41.37%) are labeled as medium-performing, and 569 (28.78%) are labeled as poorly-performing merchants. To ascertain the reliability of the labels in providing insights into the future performance of merchants and to assess the potential influence of merchant location and customers’ socio-demographic factors, such as income and wealth, on the assigned labels, we conduct the following analyses.

#### Label indication

To investigate if the defined labels are able to distinguish between well-performing and poorly-performing merchants, and provide insights into merchants’ performance in the longer terms, we compare the merchants that possess similar magnitudes of revenue, number of customers, and transaction counts in the first period, but are labeled oppositely (i.e., poorly-performing vs. well-performing) bringing into account their performance in the second period.

To this end, we first convert the revenue, transaction count, and number of unique customers of merchants during the first period (first 6 months) into quartiles (i.e., Q1, Q2, Q3, and Q4). Table 3 illustrates an example of the resulting data table structure by providing a random sample of rows. Then we choose the merchant pairs from the same MCC and the same quartiles of revenue, transaction count, and the number of unique customers in the first period.

**Table 3:** Example table of merchants’ revenue, transaction count, and unique customers count, presented in quartiles.

$\text {ID}^{\text {m}}$	$\text {L}^\text {m}\text {(t+1)}$	$\text {Q}_\text {R}^\text {m}\text {(t)}$	$\text {Q}_\text {C}^\text {m}\text {(t)}$	$\text {Q}_\text {N}^\text {m}\text {(t)}$
119811014	well-performing	Q4	Q3	Q3
119811011	poorly-performing	Q4	Q3	Q3
119811067	medium-performing	Q2	Q1	Q1
119811051	medium-performing	Q2	Q1	Q1
119811017	well-performing	Q1	Q3	Q3
119811002	poorly-performing	Q1	Q3	Q3
119811051	well-performing	Q1	Q2	Q3
119811018	poorly-performing	Q1	Q2	Q3

We only keep the pairs that are labeled oppositely (i.e., well-performing and poorly-performing) based on their second-period performance indicators. Those merchant pairs are the ones that: (1) fall into the same quartiles of merchant revenue, transaction count, and distinct customer count, and (2) one of them is labeled as well-performing and the other is labeled as poorly-performing. There are 11,813 pairs of merchants in our dataset that satisfy both conditions. Table 4 provides an example of the data table resulting from using the information presented by Table 3 as input.

**Table 4:** Merchant pairs with the same quartiles of revenue, transaction count, and unique customers count but opposite labels.

$\text {ID}^{\text {m1}}$	$\text {ID}^{\text {m2}}$	$\text {L}^\text {m1}\text {(t+1)}$	$\text {L}^\text {m2}\text {(t+1)}$	$\text {Q}_\text {R}^\text {m1,m2}\text {(t)}$	$\text {Q}_\text {C}^\text {m1,m2}\text {(t)}$	$\text {Q}_\text {N}^\text {m1,m2}\text {(t)}$
119811014	119811011	well-performing	poorly-performing	Q4	Q3	Q3
119811017	119811002	well-performing	poorly-performing	Q1	Q3	Q3
119811051	119811018	well-performing	poorly-performing	Q1	Q2	Q3

Next, using the ordinary least squares method, for each merchant we compute three fitted line slopes taking into account their monthly revenue, transaction count, and number of unique customers as dependent variables and month numbers as the independent variable. Those slopes can provide an overall indication of a merchant’s performance considering each variable over 12 consecutive months. Equation (12) shows the closed-form expression of the regression model where $\beta _1$ denotes the value we use as fitted line slope in our analysis.

12 $$\begin{aligned} \begin{aligned}{}&Y = \beta _0 + \beta _1 X + \epsilon \\&Y = (Y_1,...,Y_{12})^\intercal ,\; X = (1,...,12)^\intercal ,\; \text {and} \; \epsilon = (\epsilon _1,...,\epsilon _{12})^\intercal \end{aligned} \end{aligned}$$

**Figure 2:**
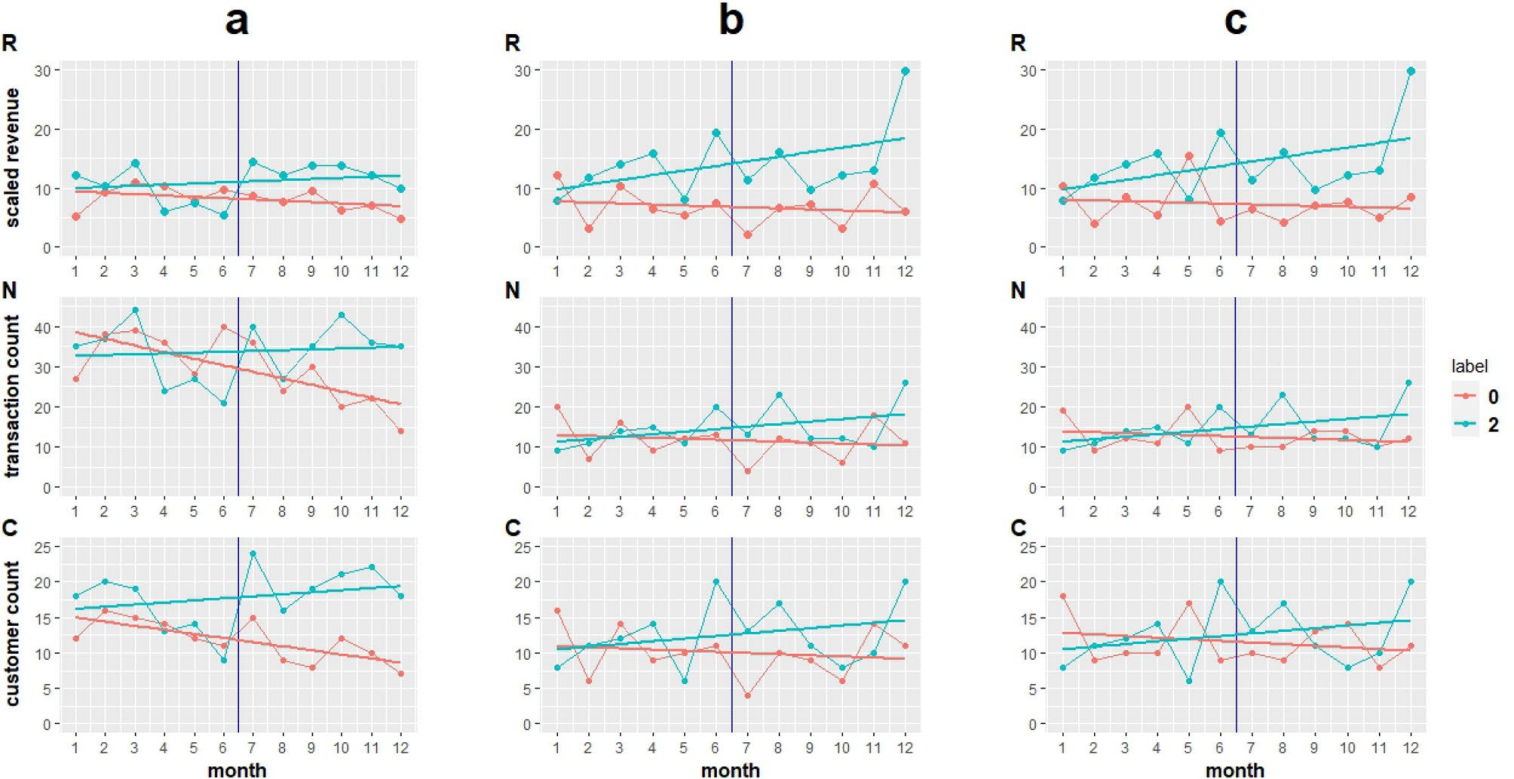
Each sub-figure (i.e., a, b, and c) depicts three time series plots for different merchant pairs with the same quartiles of revenue, transaction count, and unique customers count in the first period, but opposite labels. In each sub-figure the time series plots show monthly revenue (top: R), monthly transaction count (middle: N), and monthly unique customer count (bottom: C) including the OLS regression line for each merchant over the course of 1 year. In the figure legend, 0 corresponds to merchants labeled as poorly-performing, and 2 corresponds to those labeled as well-performing.

Figure 2 shows the time series plots for three instances provided in Table 4. Each sub-figure (i.e., 2a, b, and c) depicts time series plots of a merchant pair’s monthly revenues (i.e., 2a$_\text {R}$, 2b$_\text {R}$, and 2c$_\text {R}$), monthly transaction counts (i.e., 2a$_\text {N}$, 2b$_\text {N}$, and 2c$_\text {N}$), and numbers of their monthly distinct customers (i.e., 2a$_\text {C}$, 2b$_\text {C}$, and 2c$_\text {C}$) over 12 months of historical credit card transaction records. The vertical blue line in each plot splits the time-frame into two periods of 6 months as we used for labeling. All pairs are chosen from the same quartile of customer count, transaction count, and revenue in the first period and may have similar trends during the first 6 months. In contrast, each merchant pair have opposite labels (i.e., well-performing vs. poorly-performing) according to their performance indicators values in the subsequent period. As illustrated in Figure 2, it is evident that each merchant pair have a comparable performance during the first period, but their performance is significantly different during the second period. For visualization purposes, the fitted lines representing merchants labeled as well-performing are depicted in green, while the fitted lines for those labeled as poorly-performing are displayed using red color. The lines in each chart are the OLS fitted lines and their slope is equal to $\beta _1$ in Eq. (12). Those lines provide insights into merchants’ overall performance trend considering each of the three factors, namely: revenue, transaction count, and distinct customer count over the course of 12 months.

In the subsequent step, we compare the $\beta _1$ values for each pair of merchants. For each slope pair, if the merchant labeled as well-performing has a higher slope, we assign 1 to the slope indicator and otherwise assign 0, and then sum the three resulting indicators. If the well-performing merchant has higher $\beta _1$ values in all three indicators, the sum will be equal to 3. Conversely, if the well-performing merchant has lower values in all three indicators, the sum will be equal to 0. The closed-form expressions of these calculations are presented in Eqs. (13–16).

13 $$\begin{aligned}{} & {} {I_{{\beta }_{1}}^{R}} \rightarrow {\left\{ \begin{array}{ll} 1\quad if\quad \beta _{1}^{R_{well-performing}}\; \ge \; \beta _{1}^{R_{poorly-performing}} \\ 0\quad otherwise \end{array}\right. } \end{aligned}$$

14 $$\begin{aligned}{} & {} {I_{{\beta }_{1}}^{C}} \rightarrow {\left\{ \begin{array}{ll} 1\quad if\quad \beta _{1}^{C_{well-performing}}\; \ge \; \beta _{1}^{C_{poorly-performing}} \\ 0\quad otherwise \end{array}\right. } \end{aligned}$$

15 $$\begin{aligned}{} & {} {I_{{\beta }_{1}}^{N}} \rightarrow {\left\{ \begin{array}{ll} 1\quad if\quad \beta _{1}^{N_{well-performing}}\; \ge \; \beta _{1}^{N_{poorly-performing}} \\ 0\quad otherwise \end{array}\right. } \end{aligned}$$

16 $$\begin{aligned}{} & {} { I_{{\beta }_{1}}^{S} = I_{{\beta }_{1}}^{R} + I_{{\beta }_{1}}^{C} + I_{{\beta }_{1}}^{N}} \end{aligned}$$

Table 5 summarizes the result of this analysis. It is evident that in more than 90% of the pairs, the merchant labeled as well-performing demonstrates superior long-term and overall performance across all three factors: revenue, transaction count, and the number of unique customers. These results affirm the robustness and high accuracy of our proposed labeling approach in effectively distinguishing merchants based on their comprehensive performance measured by three distinct objective criteria.

**Table 5:** Number and percentage of merchant pairs by their sum indicators.

Sum Indicator ($I_{\beta _{1}}^{S}$)	Merchant pair count	Merchant pair percentage
0	160	1.35%
1	239	2.02%
2	673	5.69%
3	10,741	90.92%

#### District level analyses

In this section, we conduct three statistical analyses to explore potential relationships between merchants’ performance and their geographical location (i.e., district).

##### Correlation analysis

Initially, we calculate the proportion or share of each performance group (i.e., well-performing, medium-performing, and poorly-performing) within each district based on their respective labels. These shares represent the probability of a merchant belonging to each performance class within a particular district. Furthermore, we compute the relative ratios of performance class pairs for each district. Subsequently, we conduct a correlation analysis to examine the potential associations between the probabilities and relative ratios of performance classes with the population and average household income of their corresponding districts. The results reveal no correlations between the performance class of merchants and the population size or income level of the residents in the districts where they are located (correlation table is provided in “Supplementary Information”).

##### Chi-squared test

This test is performed between district ids as categorical variable (identification numbers) and performance class probabilities converted into categorical variables. This type of test is valid here as the sample size is small (33 districts) and the contingency table test is reasonable. Based on the results ($\chi ^{2}(2560, \, n=99) \, = \, 2629, \, p = 0.167$), there are no observed dependencies between the performance class probability distributions and relative ratios with the district ids indicating that within our dataset and based on the defined labels, a merchant’s performance is independent of the district in which it is situated.

While based on Eq. (17) the inequality value can range from 0 (perfectly equal) to 1 (fully unequal), the histogram and the distribution range presented in Table 6 reveal that, in district-level distributions, the inequality scores tend to concentrate towards lower values, suggesting a trend towards reduced inequality. It is important to note that the maximum inequality index value obtained here (i.e., 0.388), is even less than 40% of the maximum possible value. Furthermore, the other statistical measures, such as median, mean, and interquartile range, further support the observed trend of the distribution favoring low inequality values.

In conclusion, the findings obtained from the three aforementioned analyses validate that there is no discernible dependency or substantial association between the defined performance labels for merchants and the districts in which they are situated.

##### Label distribution inequality analysis

For this analysis, we use the formulation presented in Eq. (17) to measure the inequality^52^ in performance class labels’ distributions within and among districts. This equation formally represents the inequality of labels’ distribution in district *i* (denoted by $Inequality_L^i$) as a function of shares of the three labels in that district (denoted by $p_{L_{k}}^{i}$).

17 $$\begin{aligned} \begin{aligned}{}&Inequality_L^i = \frac{3}{4} \times \sum _{k=1}^{3} |p_{L_{k}}^{i} - \frac{1}{3}| \\&(Inequality_L^i \in [0, 1]) \end{aligned} \end{aligned}$$

**Figure 3:**
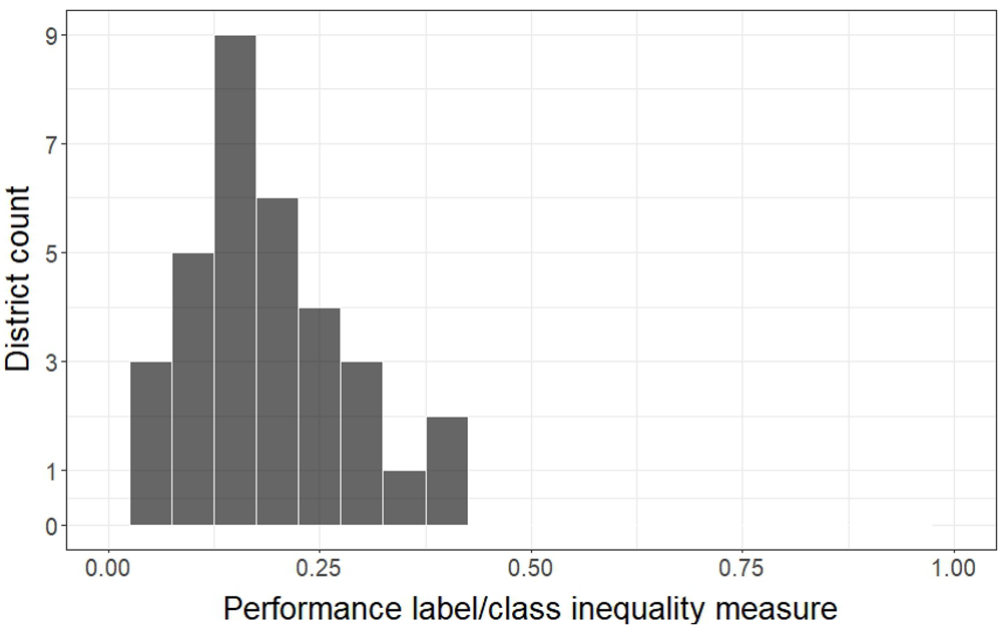
District level label inequality histogram.

Since by definition, there are 3 performance labels ($k \in {1, 2, 3}$), then the classes in a totally equal distribution should have the same share of the merchants in a district, that is equal to one third (i.e., $\frac{1}{3}$) share for each class, which results in 0 inequality. Moreover, a completely unbalanced distribution happens when the share of one particular class is equal to 1 and the other two classes have 0 shares. This distribution will yield the highest possible inequality value, which is equal to 1. Table 6 provides the basic statistics of the label inequality measures at a district level, while Fig. 3 illustrates the corresponding histogram.

**Table 6:** Basic statistics of the label inequality measures at districts level.

Range	Mean	Median	Standard deviation	Inter-quartile range
[0.042 , 0.388]	0.188	0.164	0.094	0.11

#### Customer level analysis

To explore potential dependencies between a merchant’s performance and the income level of its customers, we categorize the mean and median income of all merchants’ customers into quartiles based on their respective distributions. This results in four categories (i.e., quartiles) for merchants based on the mean and median income of their customers. Subsequently, we conduct two chi-squared tests to examine the relationship between a merchant’s performance label and the quartiles of its customers’ mean and median income. The results of these tests indicate no significant dependencies between a merchant’s performance class and the mean ($\chi ^{2}(6, \, n=1977) \, = \, 5.2951, \, p = 0.506$) or median ($\chi ^{2}(6, \, n=1977) \, = \, 2.8613, \, p = 0.826$) income of its customers. Additional analysis and the corresponding results regarding the customer features and their role in generating the merchant network of study are presented in the “Supplementary Information”.

Based on the findings obtained from both the district-level and customer-level analyses, it can be concluded that the defined labels are independent of and unbiased towards the locations of merchants or the socio-economic status of their customers. This reaffirms the robustness and reliability of the labeling approach proposed and used in the study.

### Predictive analysis

For each merchant, we gather 25 features to serve as inputs for the machine learning models. Out of these 25 features, 5 are based on the merchant’s information, 3 are related to revenue, 11 are obtained from customer data, and 6 are extracted from the proposed network structure. The obtained merchant network consists of 2,011 nodes and 217,422 edges as one strongly connected component. “Supplementary Information” provides additional details concerning the network and node features. In addition to the 6 features directly obtained from the network structure, we generate a feature vector with 128 dimensions for each merchant (node) using the node2vec method.

Then we use different combinations of the feature sets as inputs for the four selected machine learning models. Table 7 summarizes the results for the classifiers we include in our analyses. Among those, Random Forest (RF) performs better than the other classifiers in most cases. This is in line with the previous findings that show tree-based models, and in particular RF, perform better in multi-class performance prediction tasks^53–55^.

The computational results shown in Table 7 indicate that the performance of the network-based feature set is comparable to and almost as good as other feature sets with all classifiers. Moreover, in certain scenarios, the models utilizing feature vector representation (i.e., node2vec) of merchants within the suggested network demonstrate superior performance compared to those that solely use customer-based features. Nevertheless, the node2vec features exhibit no improvement over the network-based features, potentially due to information loss during the embedding process.

It is worth emphasizing that the inclusion of network-based features alongside the conventional feature sets enhances prediction accuracy. This suggests that certain signals, which are not captured by revenue-based and customer-based information, can be captured through features associated with a merchant’s position within the proposed merchant network.

**Table 7:** AU-ROC produced by four classifiers using different combinations of input feature sets.

Feature set	NB	SVM	LR	RF
(A) Revenue	0.565	0.557	0.587	0.586
(B) Customer	0.558	0.561	0.579	0.571
(C) Network	0.561	0.556	0.575	0.579
(D) node2vec	0.537	0.556	0.567	0.572
(A) + (B)	0.566	0.566	0.588	0.591
(A) + (B) + (C)	0.569	0.567	0.598	0.609

**Figure 4:**
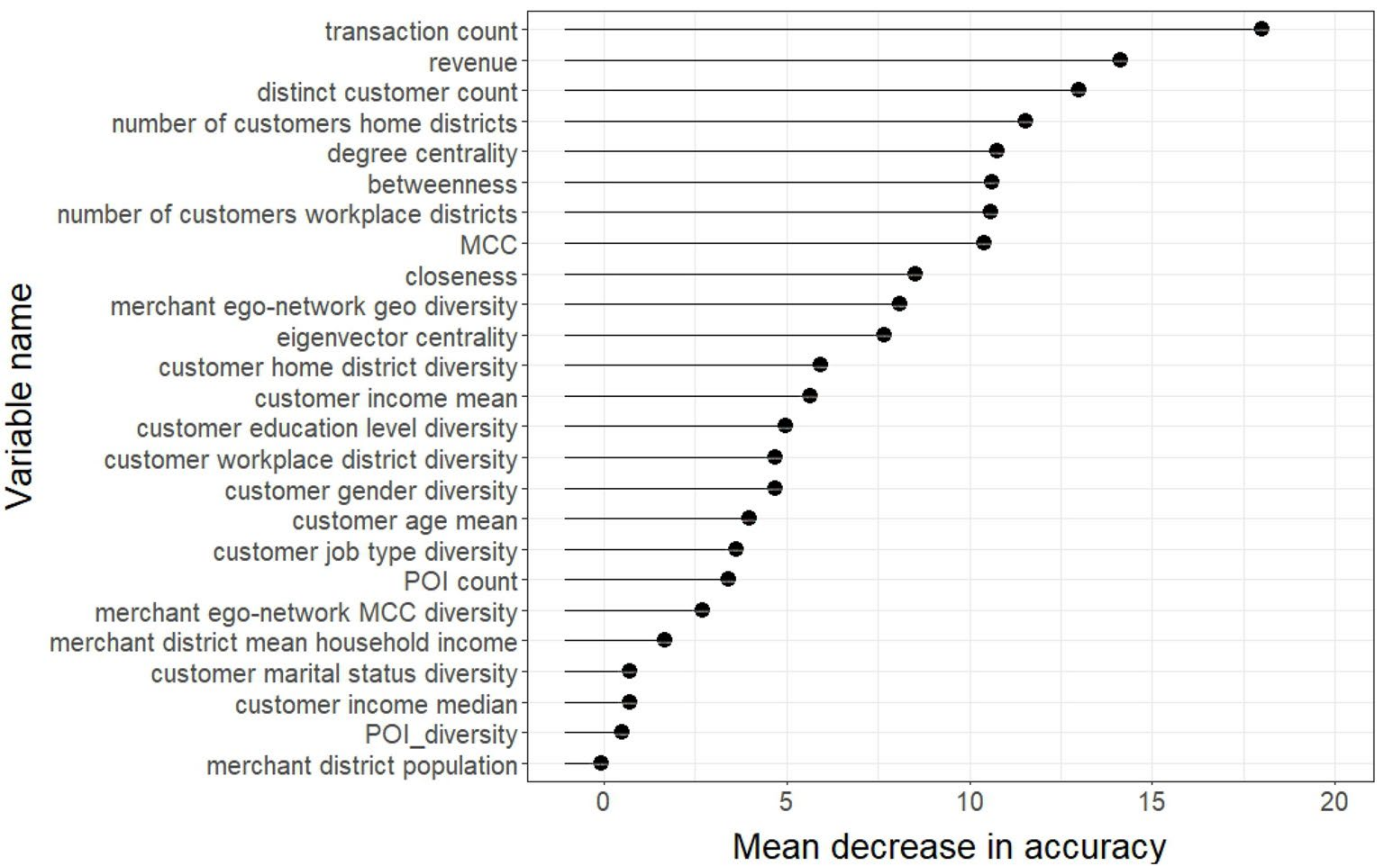
Feature importance ranking for Random Forest classifier based on the mean decreasing values of accuracy after randomly permuting the relevant feature. A higher decreasing value of a feature indicates more contribution.

In order to demonstrate the impact of each feature on prediction accuracy, we perform a feature importance analysis using the mean decrease in accuracy. This analysis reveals the reduction in accuracy that occurs when a particular feature is absent. Figure 4 displays feature importance rankings for the RF classifier in accordance with the mean decrease in the classifier’s prediction accuracy when a feature is removed after a random permutation. With the mean decrease in accuracy measures, financial features such as transaction count, revenue, and the number of unique customers, occupy the top ranks as they signify their role in accurate predictions. Among the network-based features, degree centrality, followed by betweenness and closeness centrality scores, cause the greatest deficits in accuracy. Notably, among the top ten important variables based on the mean decrease in accuracy, four belong to the network-based features.

The results of analyses shown in Table 7 and Fig. 4 demonstrate that in some prediction results as well as the feature importance ranking cases, some features from different feature sets possess close performance levels. This similarity can be the result of high correlation between the features from different feature sets, which also indicates that those features could carry similar signals and information about the merchants but obtained in different ways. For instance, features such as node degree, revenue, and the number of customers, originating from different feature sets, demonstrate significant pairwise correlations and closely align in their importance ranking. The correlation table of the features used in this study is available in “Supplementary Information”.

In addition, we employ two different methods to further explore the relationship between labels and extracted features in our study. Firstly, we conducted principal component analysis (PCA)^56^ on the originally extracted features, as presented in Table 2. Secondly, we utilize T-distributed stochastic neighbor embedding (t-SNE)^57^ on the node2vec features. These methods were employed to visualize the placement of merchants in a two-dimensional (2D) space. The resulting 2D plots do not exhibit any discernible distribution or clustering patterns for merchants based on their performance classes. For more comprehensive information, including detailed plots, please refer to the “Supplementary Information”.

### Privacy implications

Within a highly competitive environment, the proprietary financial information of merchants assumes a heightened level of sensitivity. Consequently, the custodians of such data (e.g., banks), exercise caution in divulging such confidential information to external entities. However, financial institutions and investors find themselves compelled to evaluate the stability of merchants and predict their future performance in order to make informed determinations regarding business loans and investments. As a result, accessing this confidential information becomes imperative for these stakeholders. However, given the context of data sharing, safeguarding merchants’ revenue- and customer-related information from unauthorized disclosure assumes paramount importance and is of utmost priority.

Our methodology offers a twofold advantage. Firstly, the network-based features generated through our approach exhibit prediction accuracy on par with conventional feature sets. Secondly, these features are presented in a tabular format, enhancing privacy beyond that offered by merely anonymizing raw financial records. This unique property enables the establishment of a more secure and expeditious data-sharing process with third-party organizations. Such a mechanism holds immense potential for enhancing data privacy while facilitating efficient collaboration and knowledge exchange.

The nature of network-based features, which primarily reveal the interconnectedness within the merchant network, presents a challenge in directly inferring and estimating customer- and revenue-related information in the absence of raw data or relevant statistical measures (e.g., average spending per customer transaction) which are never released by a financial institution that owns the raw data. Nevertheless, due to the significant correlation between certain financial or customer-related variables (e.g., revenue and the number of customers) and network-based features (e.g., node degree), there is a possibility of inferring sensitive information, such as revenue ranking and comparison.

To address this issue, the network data owner has the option to generate node2vec features locally and share them with third parties for the downstream task, specifically merchant performance classification. This approach is justifiable, as demonstrated by the outcomes presented in Table 7, wherein node2vec features exhibit comparable results to network-based features while ensuring a higher level of privacy.

While there exist adversarial attack methods aimed at reconstructing the graph from the node embeddings of the original graph, it is important to note two significant limitations. Firstly, these methods are unable to fully recover the original graph, thereby compromising the reliability of the reconstructed network in providing accurate insights about merchants. Secondly, data holders can effectively mitigate privacy risks associated with integrating node embeddings for downstream analysis by employing suitable defense mechanisms.

In the realm of defense mechanisms, two widely employed approaches are perturbation of the node2vec matrix, and generating embeddings in lower dimensions^58,59^. While these methods effectively mitigate inference attacks’ accuracy, they come at the expense of degrading the accuracy of merchant performance prediction tasks. To tackle this issue, one can employ a tentative defense mechanism that involves an iterative process of removing the least significant feature vector (i.e., column) from the node2vec matrix until no substantial changes are observed in the classification accuracy. This technique is both straightforward and effective, while ensuring that our proposed approach does not compromise the accuracy of the downstream classification task.

## Discussion

Although SME merchants contribute significantly to employment and economic activity, historical data indicates that only approximately 50% of them manage to sustain their businesses beyond the initial five years. Economic downturns and financial crises can further compound the challenges faced by SMEs, leading to a significant negative impact on the business continuity of the majority of SME merchants.

Furthermore, SME merchants heavily rely on business financing and external investments. Given the high failure rate among SMEs, banks and other financial institutions face a dire need for effective models and tools to assess the current state and predict the future performance of their clients. Complicating matters, the privacy concerns surrounding SMEs often result in the unavailability of their financial data and reports for sharing with external entities, and therefore, the data-driven methods and tools become ineffective and useless when the necessary data is not accessible. This fact underscores the significance and urgency of developing methods for sharing information swiftly and securely, without compromising privacy.

To tackle these challenges, one potential solution is to utilize credit card transaction information, which is accessible to banks and select financial institutions. In this study, we offer insights to banks and financial institutions regarding the utilization of credit card transaction data in order to enhance the accuracy of predicting a merchant’s future performance. We present a new approach that involves constructing a merchant network, based on customer co-purchase patterns, derived from credit card transaction data. This network structure possesses distinct characteristics that enable the extraction of various signals, including four different centrality measures. These measures indicate the position of a specific merchant within the broader network of merchants and its level of connection to other merchants, whether from the same or different business categories within its ego network. Notably, we discover that these features provide sufficient signals to predict a merchant’s future business performance. This approach fundamentally differs from the commonly employed methods found in current state-of-the-art techniques.

Our computational results show that the network-based features are capable of revealing new information about a merchant’s performance level that is on par with more straightforward measures such as demographic characteristics and composition of customers visiting the merchant, and financial indicators of the merchant such as total revenue or the number of transactions generated. We find that, with the use of effective machine learning modeling techniques demonstrated in this study, analysts can more accurately predict the future performance level of merchants using the features extracted from the proposed network. Such predictions are important for financial institutions because a major portion of their clients are SMEs, which may exhibit erratic financial behavior and performance, and may not always disclose the details of their financial records. Considering the substantial number of SME merchants and their elevated default rate, even marginal improvements in prediction accuracy can significantly aid financial institutions in mitigating risks, particularly those with limited budget allocations.

The network-based and node2vec features not only exhibit comparable performance to conventional feature sets (i.e., revenue- and customer-based) in predicting merchants’ future performance, but they also offer a higher level of privacy when presented in a tabular format, as opposed to raw financial records. This privacy-enhancing characteristic enables the establishment of a secure and efficient information-sharing process with third-party organizations. Although inferring comprehensive financial and customer-related information solely from network-based features presents challenges, certain financial and customer indicators exhibit correlations with these features, making estimations like revenue ranking feasible. As a result, generating node2vec embeddings can facilitate safer sharing. It is worth noting that while inference attacks targeting node2vec embeddings can reconstruct parts of the original network, their success is limited. Data holders can further mitigate privacy risks associated with these attacks by employing the defense mechanisms such as those discussed in this paper.

Our study does have certain limitations that should be acknowledged. One limitation stems from our reliance on on-premise credit card transactions for constructing and validating our models. It is important to note that a portion of consumers opt for cash or online transactions, which are not included in our datasets. The number of such transactions may be non-negligible, especially in the country under study. This issue becomes even more pronounced in economies with less transparency and a higher prevalence of informal businesses that primarily operate on a cash basis. In such economies, an alternative approach could involve utilizing mobility data and visit patterns collected passively from users’ cellphones to create the merchant network^60^. However, despite these limitations, we believe that our dataset, which comprises over 2 million transactions, captures a substantial portion of the economic activity during the specified time period. Moreover, it is worth noting that there is a significant correlation between cash and card spending in larger economies^61^.

Our study possesses another limitation concerning the absence of a clear indicator or identifier within our dataset to differentiate SMEs from non-SME merchants. While certain variables such as transaction volume or revenue may suggest the classification of certain merchants as SMEs, we intentionally refrained from using such designations. As a result, our prediction models are aimed at all merchants collectively. Undoubtedly, tailoring our models specifically for predicting SME performance could have potentially enhanced their prediction accuracy. However, it is crucial to acknowledge that the network structure in which SMEs operate also involves interactions with non-SME merchants. Nonetheless, future research endeavors could focus on acquiring a dataset where SMEs can be accurately identified, allowing for the development of prediction models specifically tailored to their unique characteristics.

To summarize, our study has the following contributions. Our first contribution is anchored on the current literature about merchant risk and bankruptcy prediction using machine learning models. We build on these theories to introduce a new method in labeling merchant performance levels, which takes into account three financial objective measures considering the dynamic nature of business performance in a highly competitive environment over time.

The main contribution of this study is inspired by computational social science literature, and is built on the premise that just as we humans live our lives in networks ^23^, merchants also run their businesses in networks. Accordingly, we propose a novel approach to build a network of merchants based on their customer credit card transactions, and use the extracted features from the merchant network structure for predicting their future performance. While we show that the network-based features improve the prediction accuracy when added to the conventional revenue-based and customer-based features, they also possess a higher privacy level in comparison to the conventional feature sets, facilitating more secure and efficient data sharing among financial entities.

Our approach and methodology can further be incorporated into a decision support system or integrated with a legacy system along with exploratory visual analytics tools^62^ to be safely shared and used by various organizations including banks and financial institutions. Risk analysts and decision makers can utilize our models to rank their clients in order of decreasing estimated future risk and flag those that rank lower to take further action. The system can be configured to be a “learning” system that is continuously fed with freshly incoming transaction data and the models can periodically be re-trained to account for changes in trends and customer behavior.

Our proposed approach offers banks and financial institutions valuable new insights into predicting the future performance of their merchant clients. By leveraging network-based analysis, these institutions can enhance their ability to assess loan risks and identify investment opportunities. This study serves as an initial step toward exploring the potential of network-based methodologies for assessing merchant performance. It also highlights the possibility of developing methods that enable businesses to share information derived from data without the need to share the actual data itself.

## Supplementary Information

Supplementary Information.

## Data Availability

All pre-processed data and code necessary to replicate this study and reproduce this work are available at: https://github.com/alppboz/credit-card-research Additional analyses details and results are available in Supplementary Information of this article.
