# Supplementary material for: Predicting merchant future performance using privacy-safe network-based features
Source: Sci Rep. 2023 Jun 21;13:10073. doi: 10.1038/s41598-023-36624-0 (PMC10284870; doi:10.1038/s41598-023-36624-0)
Supplement: Supplementary file 1 — Supplementary Information. [file 41598_2023_36624_MOESM1_ESM.pdf]

Supplementary Information for:
“Predicting merchant future performance using privacy-safe network-based features”

Mohsen Bahrami, Hasan Alp Boz, Yoshihiko Suhara, Selim Balcisoy, Burcin Bozkaya, and Alex Pentland

Supplementary Notes

Datasets 1
Credit card transaction data . . . 1
Transaction table • Customer table • Merchant table
Points-of-Interest data . . . 2
Mechant Network Properties . . . 3
Additional details on feature and label analyses 5
References 6

List of Figures

1 Pair plot of customer features. . . . 3
2 Complementary cumulative distribution function (CCDF) of degree distribution and descriptive statistics of the resulting merchant network. . . . 5
3 Distribution of centrality metrics by merchant performance labels. . . . 6
4 Correlation coefficient values between label percentages and their ratio with district population and monthly average household income . . . 10
5 Correlation coefficient values for all pairs of features computed in this study. . . . 11
6 t-SNE embeddings of the merchant, customer, revenue, and network features colored by financial performance labels. 13
7 Two-dimensional visualization of merchants based on their merchant features using PCA (left) and node2vec features using t-SNE (right). Each dot represents a merchant and it color corresponds to the merchant’s performance label. . . . 13

List of Tables

1 Transaction table summary . . . 2
2 Customer table summary . . . 2
3 Twenty most frequented business categories ordered by their transaction counts in the dataset. . . . 4
4 POI groups with examples. . . . 4
5 Poisson regression analysis on the number of edges created by each customer. . . . 7
6 Percentage of the merchant network edges created by each feature group of customers, as well as the median edge created by each member within each group. . . . 8
7 Logistic Regression analysis on well-performing merchants. . . . 9
8 Alphabet codes and corresponding feature names in correlation table of Supplementary Figure 4 . . . 10
9 Alphabet codes and corresponding feature names in correlation table of Supplementary Figure 5 . . . 12

Datasets

In the following sub-sections we provide more details about the datasets used in this research.

Credit card transaction data

In this study, a large amount of credit card transaction records is used for the designed experiment. The data is a random sample of credit card transactions from a major bank in a metropolitan of an OECD country between July 2014 and June 2015. This dataset consists of three tables, namely: customer, merchant, and transaction table. In what follows, each table is described and a summary of each data table is provided. Please note that the numbers pertain to the cleaned datasets that are achieved after removing transaction records containing unknown and/or missing merchant ID or customer ID or MCC.

Transaction table

This data table contains 2,511,527 credit card transactions, each recorded as a row that contains: hashed customer ID, transaction amount, transaction date, merchant ID, and the merchant's business category. A summary of the dataset is shown in Supplementary Table 1.

Supplementary Table 1. Transaction table summary

Attribute	Value
Timeframe	12 months
Number of customers	62,194
Number of Merchants	4,507
Number of transactions	2,511,527
Average transactions per customer	40.38
Average transactions per merchant	557.25

Customer table

In this table, each row includes information pertaining to a unique customer. Each customer is defined by a hashed customer ID and for each customer 8 attributes are provided. There are 62,194 unique customer IDs in this dataset. Supplementary Table 2 provides a summary of information for the customer attributes and Supplementary Figure 1 shows the pairwise scatter plots of those features.

Supplementary Table 2. Customer table summary

Attribute	Categories and their distribution in data	Type
Customer ID	Unique hashed ID for each customer	Categorical ID number
Age	min = 19, max = 85, mean = 38.5, median = 37, IQR = 14	Numerical
Gender	Female (31.3%), Male (58.7%)	Categorical
Marital status	Single (21.4%), Married (70.8%), Divorced (4.3%), Dul (0.5%), Unknown (2.9%)	Categorical
Education level	Unknown (0.004%), Uneducated (1.2%), Elementary school (6.8%), Middle school (8.3%), High school (8.4%), Associate Degree (45.2%), Bachelor's (29.8%), Master's (2.9%), Doctoral degree (0.2%)	Categorical
Employment status	Unknown (0.1%), Unemployed (0.8%), Under work age (0.001%), Abroad (0.001%), Student (0.05%), Housewife (1.16%), Public sector (6.4%), Private sector (73.6%), Freelancer (9.8%), Retired (public sector) (4.9%), Retired (private sector) (1.93%), Retired (freelancer) (0.5%)	Categorical
Income	Monthly income as estimated by the bank	Numaerical
Home district ID	Home location district identification number	Categorical ID number
Work district ID	Workplace district identification number	Categorical ID number

Merchant table

This table includes information for 75,448 unique merchants and each merchant is identified by a unique ID, where its location district ID and business category are provided. Their business category is defined based on ISO 18245 category codes[1] referred to as merchant category code or MCC. There are 47 unique MCCs in our dataset. Supplementary Table 3 shows twenty most frequented merchant categories and their corresponding number of merchants, number of transactions, and descriptions.

Points-of-Interest data

This dataset is provided by Here.com which is a digital map production company. The dataset we use for this study, were collected via data collection vehicles moving around in every region. Additionally, they make use of several external dataset provided by local organizations to enrich their maps. We use the points of interest (POIs) data and maps for years 2015 and 2016, updated quarterly (every three months). In the dataset, POIs are grouped into twelve types. POI types with examples are shown in Supplementary Table 4.

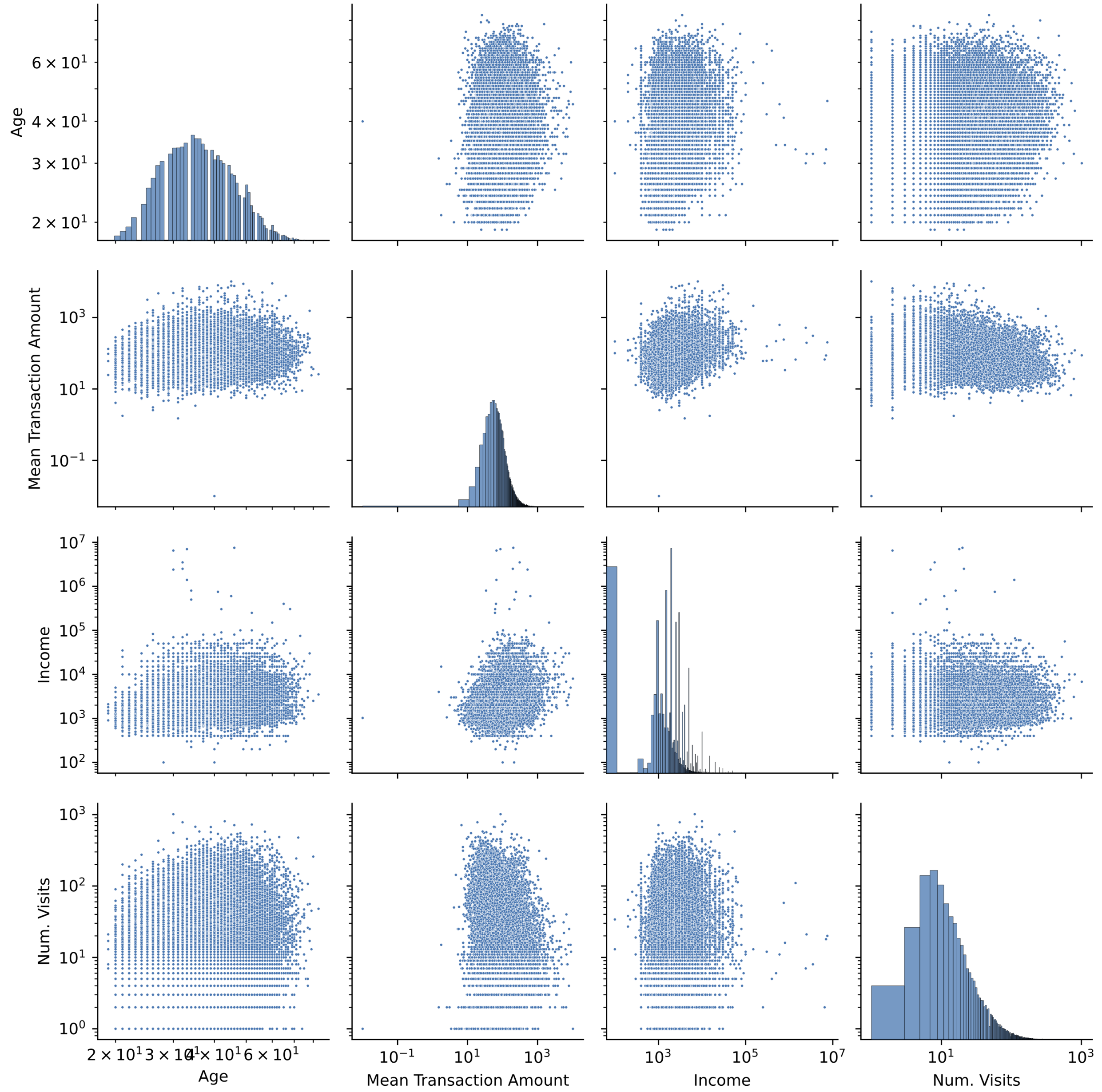

Supplementary Figure 1. Pair plot of customer features.

Mechant Network Properties

The constructed merchant network consists of a single connected component with 2,011 nodes and 217,422 edges. Supplementary Figure 2 displays the complementary cumulative distribution function (CCDF) of the degree distribution of the constructed merchant network, in addition to descriptive network properties. Overall, the network can not be considered a scale-free network, however, the tail of the distribution follows a power law with α 6.065. Moreover, the low degree of assortativity indicates that there is no association between the degrees of the connected nodes.

Supplementary Figure 3 depicts the boxplots of the node centrality metrics used in our study, grouped by the corresponding merchants' performance labels.

In order to delineate customers' role in the formation of the merchant network, we created a variable named *number of edges created*, that captures the number of edges created due to a customer's co-visit patterns. We employed customer socioeconomic attributes in a Poisson Regression model. In this setting, *education*, *Gender*, and *Marital Status* are binary variables representing, university degree or below, female or not, and married or not, respectively. The resulting coefficients are

Supplementary Table 3. Twenty most frequented business categories ordered by their transaction counts in the dataset.

MCC	Transaction count	Merchant count	Description
5411	691,957	10,376	Grocery Stores, Supermarkets
5541	382,316	4,413	Service Stations (with or without ancillary services)
5691	331,304	8,619	Men's and Women's Clothing Stores
5812	30,727	3,529	Eating places and Restaurants, and Fast Food places
5499	62,846	3,445	Misc. Food Stores, Convenience Stores, Specialty Markets
5045	68,482	548	Computers, Computer Peripheral Equipment, Software
5732	68,372	1,512	Electronic Equipment Sales
5977	42,249	1,691	Cosmetic Stores
5200	30,360	772	Home Supply Warehouse Stores
5661	25,533	2,957	Shoe Stores
5941	25,214	1,614	Sporting Goods Stores
5712	17,041	2,384	Furniture, Home Furnishings, and Equipment Stores
5942	14,046	550	Book Stores, Books, Periodicals, and Newspapers
5641	12,799	924	Children's and Infant's Wear Stores
5945	7,246	367	Hobby, Toy, and Game Shops
5992	5,889	88	Florists
5999	5,543	718	Miscellaneous and Specialty Retail Stores
5722	3,848	3,654	Household Appliance Stores
5621	3,671	1,558	Women's Ready-to-Wear Stores
5950	3,150	1,034	Glassware/Crystal Stores

Supplementary Table 4. POI groups with examples.

POI Group	Examples
Community service centers	local government offices, law courts, post offices, religious places, and wedding venues
Financial institutes	banks, exchange places, lending and financing offices
Educational institutes	including schools, universities, and private education centers
Business Centers	commercial, managerial, and other types of business offices
Entertainment places	Bars, CAFE, Disco, places for computer games, and art galleries
Shopping places	supermarkets, grocery stores, clothing, and corner shops
Restaurants	all types of eating places including fast food, Pizza, and traditional food places
Hospitals	all places which provide health services including hospitals, clinics, dentists, etc.
Parks	including public parks, public and private sport places, and gyms
Travel destinations	all tourism and lodging related places
Auto services	Places with different services for vehicles such as gas stations, car wash, & maintenance
Transportation hubs	subway and railway stations, terminals, passenger used sea ports, and bus & taxi stations

displayed in Supplementary Table 5. Education, gender, age, and marital status have a positive relationship with the number of edges created, while income and mean transaction amount displayed a negative effect on edge creation.

Apart from conducting regression analysis, we categorized the customers into two groups by utilizing each customer feature. Derived from the numeric attributes (i.e., income, age, number of transactions, and average spending per transaction), we divided the customers into two groups: those with values higher than or equal to the median, and those with values below the median. Similarly, the customers were categorized into two groups based on their binary categorical features (i.e., gender, education, and marital status). These groupings were consistent with those employed in the regression analysis. Finally, the percentage of the network edges created by each group, and the median number of edges created by each group's members were computed to provide additional insights to those indicated from the regression analysis in Supplementary Table 5. The results of the analysis are presented in Supplementary Table 6.

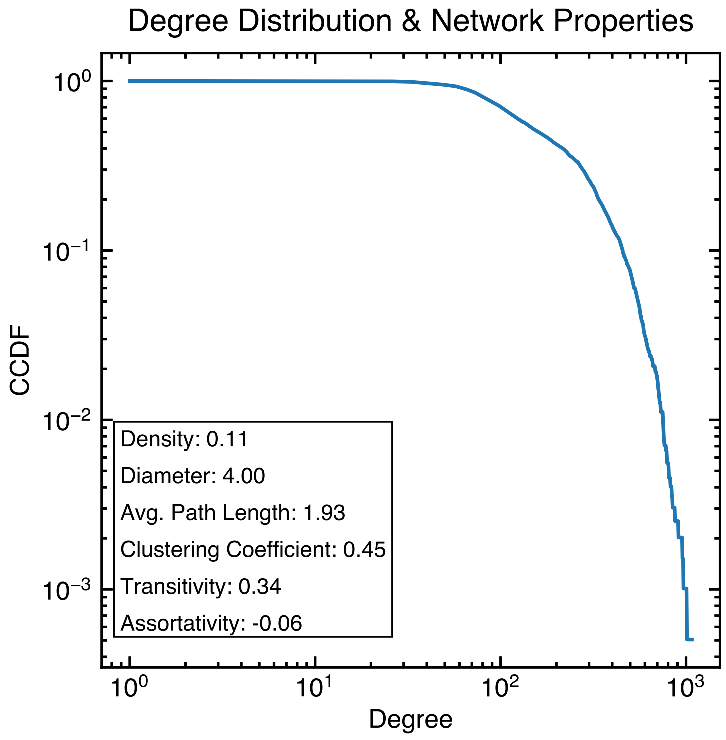

Supplementary Figure 2. Complementary cumulative distribution function (CCDF) of degree distribution and descriptive statistics of the resulting merchant network.

Additional details on feature and label analyses

In order to understand the drivers behind well-performing merchants, we conducted a logistic regression analysis based on the available feature set. First, we created a binary variable named *is merchant well-performing*, in which 1's represent well-performing merchants, while 0's represent other performance labels. In order to deal with the collinearity issue within the features, we computed the Variance Inflation Factor (VIF) for each variable and in an iterative fashion dealt with the variables with scores higher than 5. Since the centrality metrics in the network-based features are correlated, to address this problem, we applied Principal Component Analysis (PCA) on centrality metrics and represented those features with the first component. In this analysis, we also control for district and business category (MCC) variables. The resulting coefficients are displayed in Supplementary Table 7. Other than logged revenue and customer marital status entropy, none of the variables had significant coefficients. The analysis results did not provide statistically significant insights which could potentially be a result of our analytical setting which is designed for a classification problem.

Supplementary Figure 4, shows the correlation coefficients between the percentage and relative ratios of performance classes with population and average household income of their corresponding districts. Feature names and their corresponding alphabet codes are provided in Supplementary Table 8. As it is evident in Supplementary Figure 4 there are no correlations between the performance class of merchants and the population size or income of the residents of the district they are located in

Supplementary Figure 5 shows the correlation table for all features computed for this study. Diagonal cells include the alphabetical coding of each feature. Feature names and their corresponding alphabet codes are provided in Supplementary Table 9. The lower part of the table shows the numeric value of the correlation coefficient between each feature pair, and in the upper part cell, the correlation coefficients are visualized by circles, where, their radius is proportional to the magnitude of the correlation coefficient's absolute value and their sign (i.e., +/-) is demonstrated by their colors, where positive values are shown by blue circles and negative values by red circles.

In order to further investigate the relationship between labels and extracted features, we applied T-distributed Stochastic Neighbor Embedding (t-SNE) on each feature set, namely merchant, customer, revenue, and network features. Resulting two-dimensional plots are shown in Supplementary Figure 6, where each dot corresponds to a merchant and their colors represent their financial performance labels. It is evident that there are no clustering patterns for merchants based on their

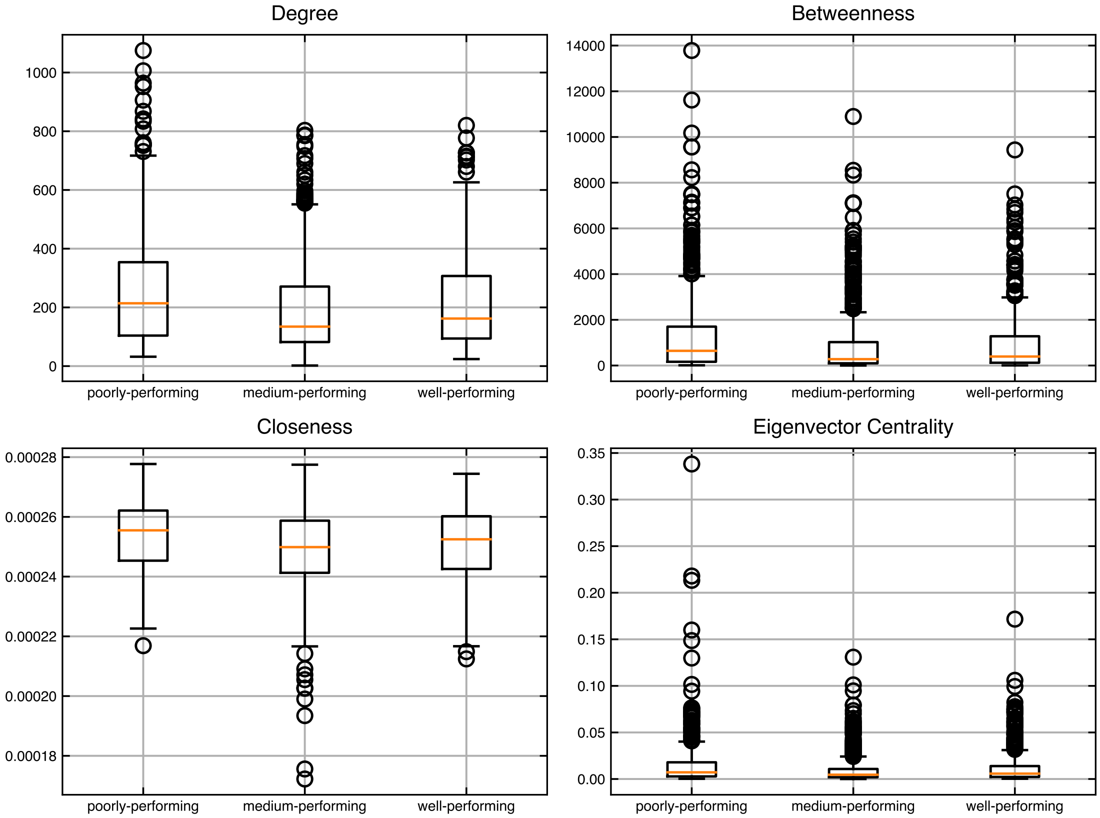

Supplementary Figure 3. Distribution of centrality metrics by merchant performance labels.

labels.

Additionally, we conducted PCA on the originally extracted features and t-SNE on the node2vec features. We used these methods for visualizing merchants in a two-dimensional space. The resulting two-dimensional plots are presented in Supplementary Figure 7, where each dot corresponds to a merchant and their colors represent their labels. The patterns indicate that there are no clustering patterns for merchants based on their labels in both sub-figures.

References

1. ISO 18245 Merchant Codes. https://www.iso.org/standard/33365.html. Accessed: 2023-01-14.

Supplementary Table 5. Poisson regression analysis on the number of edges created by each customer.

	Dependent variable:
	Num. of Edges Created
Education	0.410***
	(0.003)
Gender	0.060***
	(0.003)
log(Income)	−0.004***
	(0.0005)
Age	0.006***
	(0.0002)
Marital Status	0.281***
	(0.003)
log(Mean Transaction Amount)	−0.308***
	(0.002)
Constant	3.428***
	(0.010)
Observations	38,192
Log Likelihood	−638,292.000
Akaike Inf. Crit.	1,276,598.000
Note:	*p<0.1; **p<0.05; ***p<0.01

Customer feature	Group	Percentage of all	Percentage edges created	Median edge created
Income	Above median	54.2%	60.0%	6
	Below median	45.8%	40.0%	6
Age	Above median	53.6%	56.2%	6
	Below median	46.4%	43.8%	6
Number of transactions	Above median	51.1%	85.0%	15
	Below median	48.9%	15.0%	3
Average spent	Above median	50.0%	42.5%	6
	Below median	50.0%	57.5%	10
Education	University degree	29.7%	37.1%	10
	Below	70.3%	62.9%	6
Gender	Female	32.2%	34.0%	6
	Male	67.8%	66.0%	6
Marital status	Married	72.9%	76.0%	6
	Not married	27.1%	24.0%	6

Supplementary Table 6. Percentage of the merchant network edges created by each feature group of customers, as well as the median edge created by each member within each group.

Supplementary Table 7. Logistic Regression analysis on well-performing merchants.

	Dependent variable:
	Is Merchant Well-Performing
log(Revenue)	−0.360***
	(0.089)
log(POI Count)	0.040
	(0.056)
POI Diversity	0.065
	(0.162)
Customer Median Age	0.028
	(0.025)
log(Customer Median Income)	0.396
	(0.300)
Customer Gender Entropy	0.359
	(0.526)
Customer Job Type Entropy	−0.390
	(0.273)
Customer Education Entropy	0.311
	(0.340)
Customer Marital Status Entropy	−0.834***
	(0.319)
Merchant Ego-Net MCC Entropy	0.016
	(0.202)
Merchant Ego-Net GEO Entropy	−0.001
	(0.062)
Centrality PCA	−2.047
	(2.620)
Observations	1,977
Log Likelihood	−1,144.396
Akaike Inf. Crit.	2,382.792
Note:	*p<0.1; **p<0.05; ***p<0.01

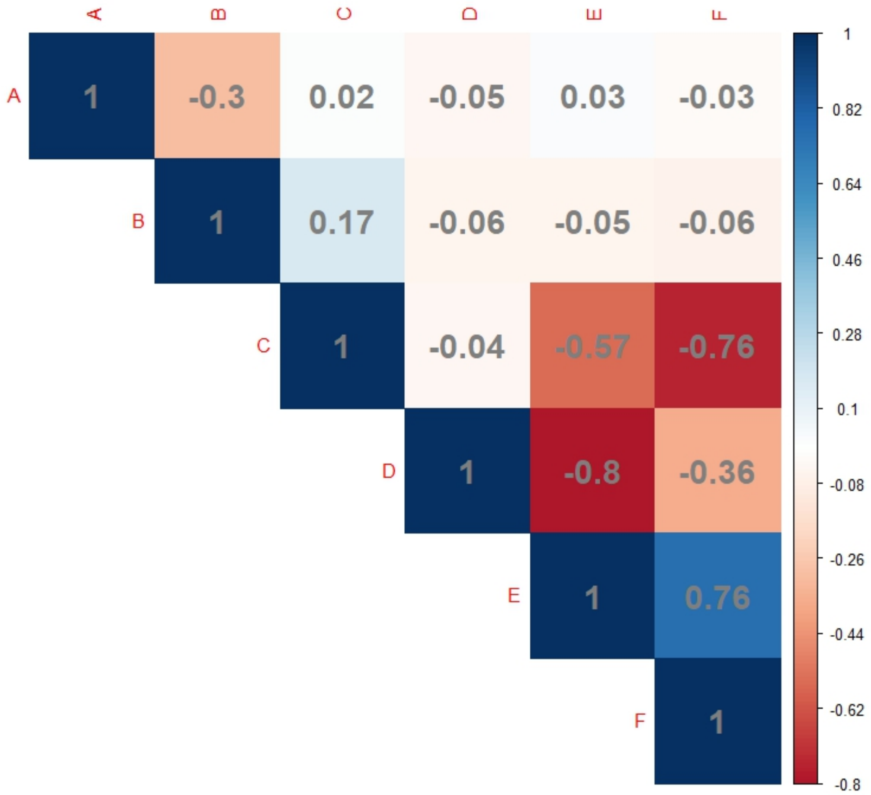

Supplementary Figure 4. Correlation coefficient values between label percentages and their ratio with district population and monthly average household income

Supplementary Table 8. Alphabet codes and corresponding feature names in correlation table of Supplementary Figure 4

Feature Name	Alphabet code
merchant district population	A
merchant district average household income	B
percentage labeled as poorly-performing	C
percentage labeled as medium-performing	D
percentage labeled as well-performing	E
well-performing to poorly-performing class percentage ratio	F

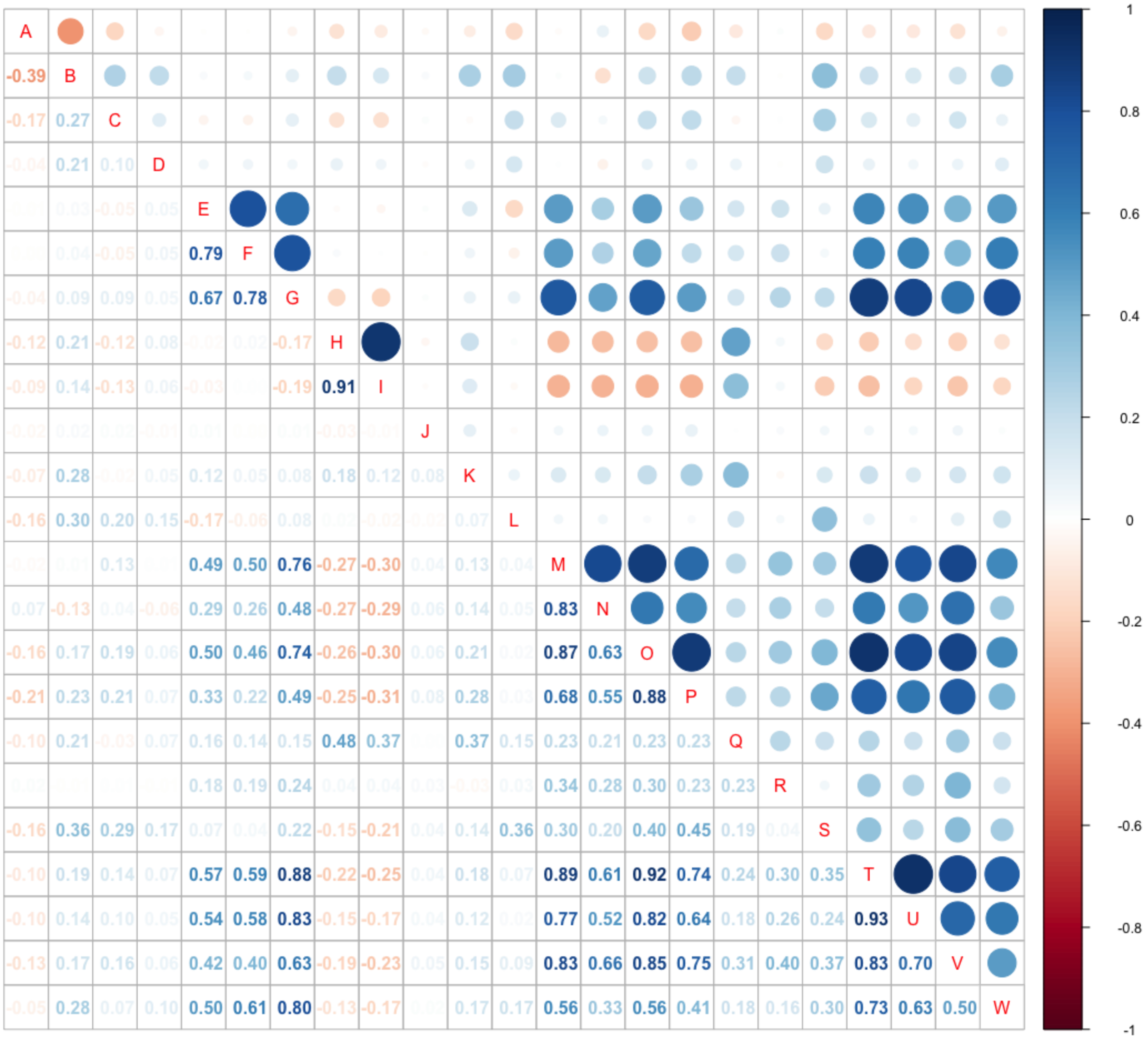

Supplementary Figure 5. Correlation coefficient values for all pairs of features computed in this study.

Supplementary Table 9. Alphabet codes and corresponding feature names in correlation table of Supplementary Figure 5

Feature Name	Alphabet code
merchant district population	A
merchant district average household income	B
merchant in buffer POI count	C
merchant in buffer POI diversity	D
period revenue	E
period transaction count	F
period distinct customers count	G
customer age mean	H
customer age median	I
customer income mean	J
customer income median	K
customer gender entropy	L
number of customers workplace districts	M
customer workplace district entropy	N
number of customers home districts	O
customer home district entropy	P
customer job entropy	Q
customer education entropy	R
customer marital status entropy	S
merchant node degree	T
merchant node betweenness	U
merchant node closeness	V
merchant node eigenvector centrality	W

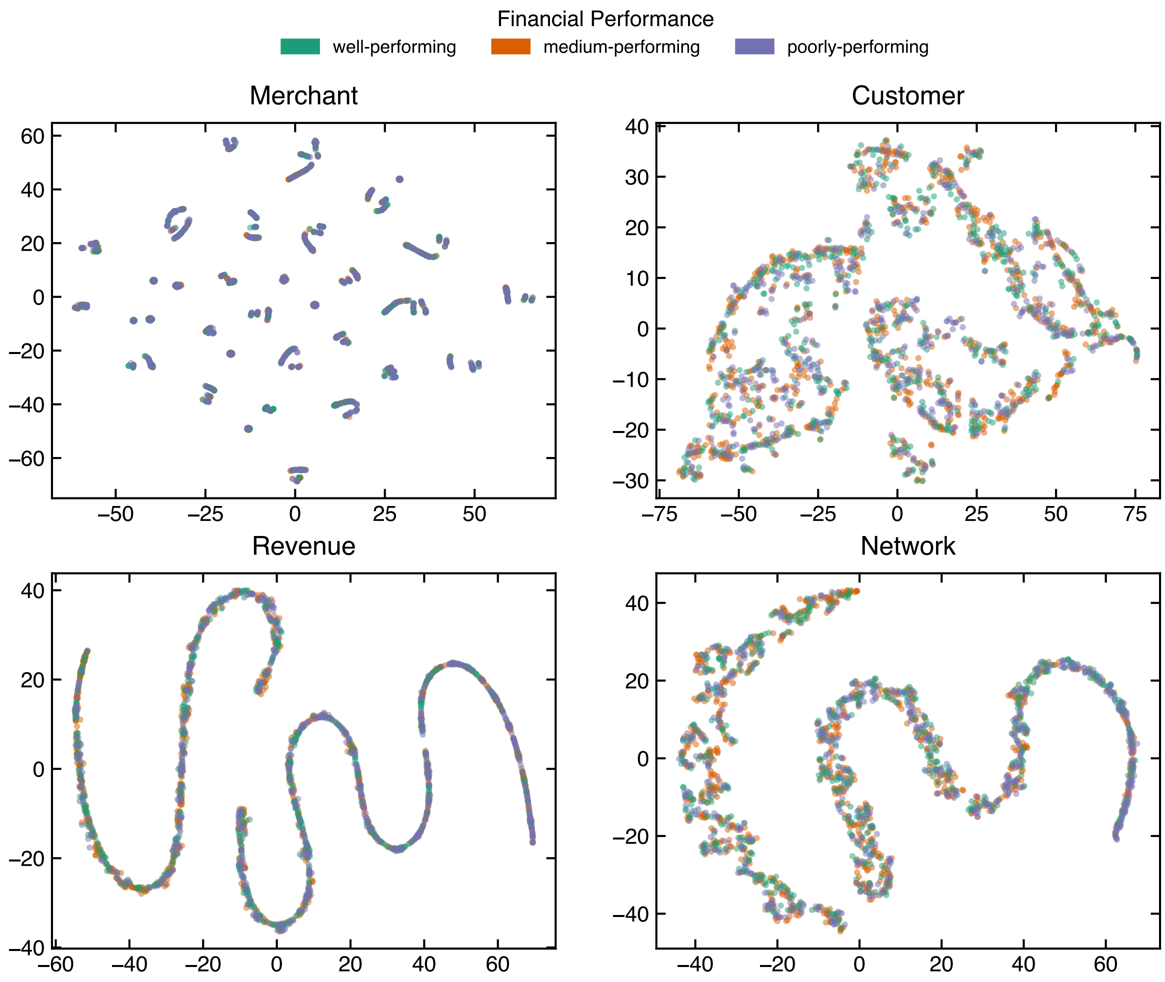

Supplementary Figure 6. t-SNE embeddings of the merchant, customer, revenue, and network features colored by financial performance labels.

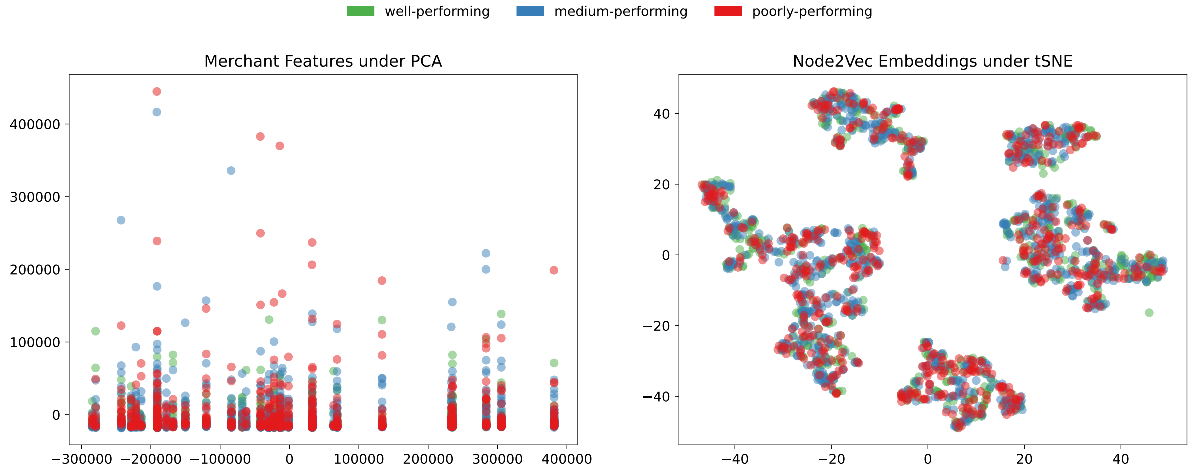

Supplementary Figure 7. Two-dimensional visualization of merchants based on their merchant features using PCA (left) and node2vec features using t-SNE (right). Each dot represents a merchant and it color corresponds to the merchant's performance label.
